# Double inferior vena cava, an uncommon but relevant anatomical anomaly in surgery for lower rectal cancer: a report of two cases

**DOI:** 10.1186/s40792-023-01738-0

**Published:** 2023-09-14

**Authors:** Mikio Kawamura, Shinji Yamashita, Hiroki Imaoka, Tadanobu Shimura, Takahito Kitajima, Yoshinaga Okugawa, Yoshiki Okita, Masaki Ohi, Yuji Toiyama

**Affiliations:** 1 Department of Gastrointestinal and Pediatric Surgery, Division of Reparative Medicine, Institute of Life Sciences, Mie University Graduate School of Medicine, Tsu, Mie 514-8507 Japan; 2 Department of Genomic Medicine, Mie University Hospital, Tsu, Mie 514-8507 Japan

**Keywords:** Double inferior vena cava, Rectal cancer, Robot-assisted surgery, Laparoscopic surgery

## Abstract

**Background:**

Double inferior vena cava (DIVC) is rare and usually detected incidentally. DIVC may be associated with several anatomical variants of the retroperitoneal and pelvic veins. These variants can pose a clinical problem during colorectal surgery. We present two patients with lower rectal cancer who also had a DIVC.

**Case presentation:**

Case 1 was a 72-year-old man with advanced lower rectal cancer (T3N0M0) who underwent robot-assisted low anterior resection after neoadjuvant therapy. A DIVC was detected on preoperative computed tomography (CT). During the operation, a presacral vein was injured while mobilizing the rectum and hemostasis could not be achieved. We converted to open surgery and packed the pelvic cavity for hemostasis. Retrospective analysis suggested the injured vein arose from an interiliac vein of the presacral pelvic venous plexus. Case 2 was a 50-year-old woman with lower rectal cancer (T3N0M0), immune thrombocytopenic purpura, and a DIVC. Although preoperative three-dimensional CT angiography showed no obvious pelvic vein abnormalities, a short course of preoperative radiotherapy was delivered to avoid lateral pelvic lymph node dissection. Chemotherapy was deferred owing to her thrombocytopenic disease. Laparoscopic abdominoperineal resection was performed meticulously to minimize bleeding and achieve rapid hemostasis. No intraoperative complications occurred.

**Conclusion:**

DIVC is often accompanied by venous malformations that may pose a problem when mobilizing the mesorectum from the retroperitoneum. Preoperative assessment of pelvic vessel anatomy using three-dimensional CT is essential in patients with a DIVC who undergo rectal surgery.

## Background

Congenital malformations of the inferior vena cava (IVC) are rare. Double IVC (DIVC) is a type of malformation which is usually found incidentally in healthy individuals during diagnostic imaging for unrelated disease [1–3]. Reported prevalence rates of DIVC range between 0.2 and 6.8% [4–6]. The IVC is formed by the confluence of the left and right common iliac veins and is usually located to the right of the aorta. DIVC is characterized by the presence of both right and left IVCs and is often accompanied by unusual venous flow through the retroperitoneal and pelvic veins such as the renal, gonadal, and iliac veins [1, 2]. Failure to recognize a DIVC and its associated abnormal venous flow patterns can lead to critical complications during urologic, gynecologic, and colorectal surgical operations. We present two patients with lower rectal cancer who harbored a DIVC and underwent surgical resection. One patient was not completely assessed before surgery and experienced massive intraoperative hemorrhage. The other was evaluated using three-dimensional (3D) computed tomography (CT) angiography and experienced an unremarkable clinical course without complications.

## Case presentation

### Case 1

**Fig. 1:**
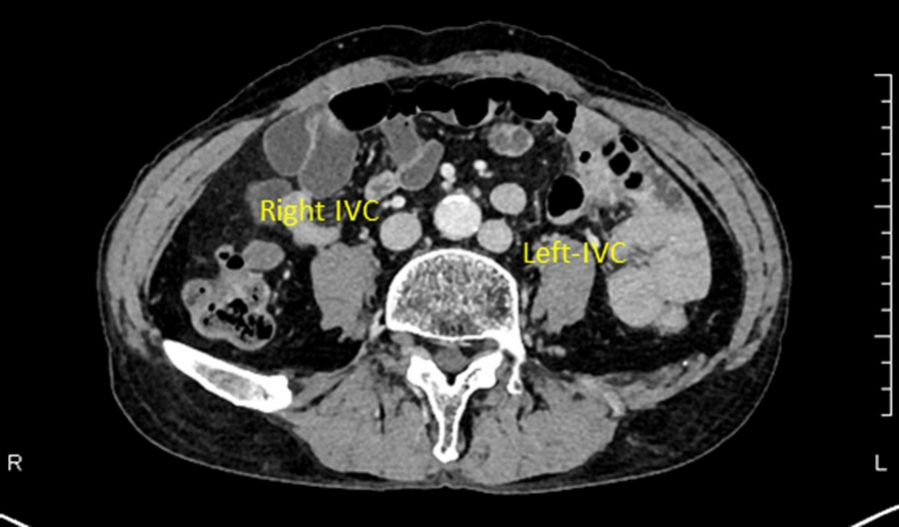
Preoperative computed tomography demonstrating a double inferior vena cava

**Fig. 2:**
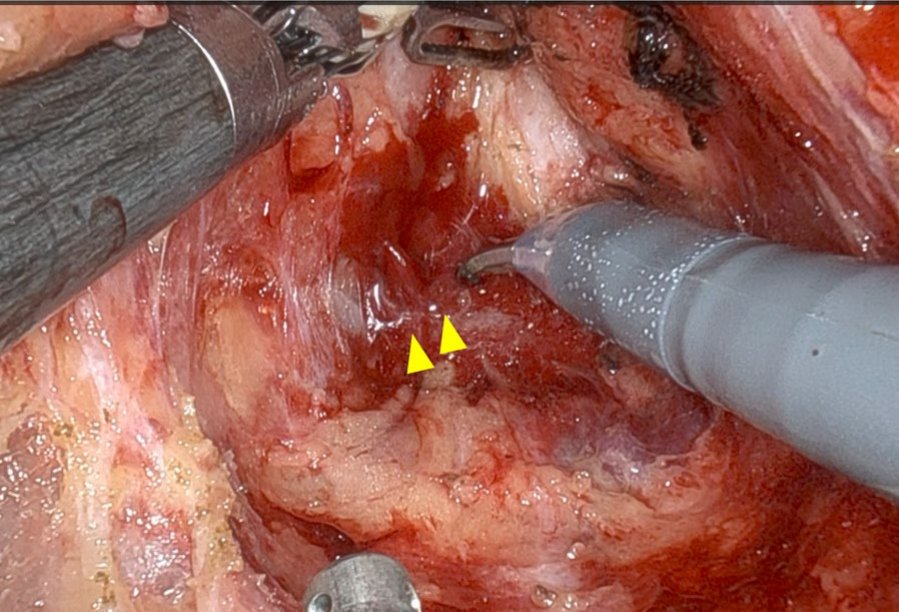
Intraoperative photograph showing the findings of presacral venous injury. After recognizing the hypogastric nerve bifurcation beyond the promontory, unusual bleeding was encountered when cutting in the sparse layer between the rectum and sacrum (arrowheads)

**Fig. 3:**
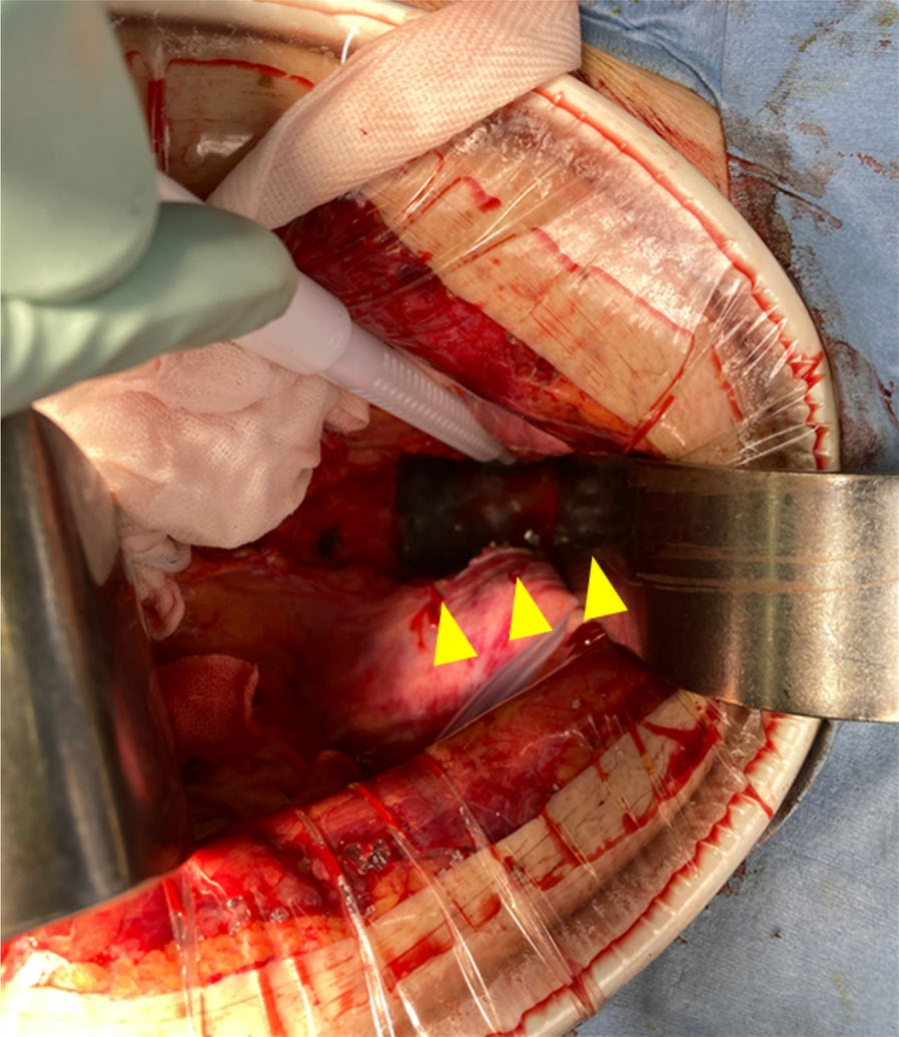
Intraoperative photograph of the second-look surgery. To reinforce the hemostasis, a sheet of polygluconic acid (arrowheads) was affixed to the sacrum and fibrin glue was sprayed

A 72-year-old man with advanced lower rectal cancer was referred to our department. Digital rectal examination revealed a tumor located in the anterior wall approximately 5 cm from the anal verge. Colonoscopy showed a type 2 tumor. Biopsy findings were consistent with well differentiated adenocarcinoma. CT showed several swollen lymph nodes in the mesorectum; no significant lateral pelvic lymph nodes were visualized. We also appreciated a DIVC but did not recognize any other anatomical abnormalities (Fig. 1). Complementary imaging ruled out distant metastasis. On magnetic resonance imaging (MRI), the main tumor invaded beyond the muscular propria, indicating T3 disease. Based on the International Union for Cancer Control classification, cT3N0M0 lower rectal cancer was diagnosed. As neoadjuvant treatment, long course chemoradiotherapy (CRT) consisting of capecitabine and 54 Gy delivered in 30 fractions was administered followed by two cycles of capecitabine and oxaliplatin. Eight weeks after completion, endoscopy and MRI showed obvious residual cancer but no enlarged lymph nodes or distant metastasis. Robot-assisted low anterior resection was then performed using the da Vinci Xi surgical system (Intuitive Surgical Inc., Sunnyvale, CA, USA). The conventional five-port approach was used. The abdominal phase was initiated using the medial approach. After ligation of the inferior mesenteric artery and D3 lymph node dissection, the sigmoid mesocolon was separated from the retroperitoneum while sparing the gonadal vessels and ureter. During this maneuver, the left IVC was not visualized because of the hypogastric nerve plexus and retroperitoneal adipose tissue layer. In the pelvic phase, after recognizing the bifurcation of the hypogastric nerve beyond the promontory, unusual bleeding occurred when cutting in the sparse layer between the rectum and sacrum (Fig. 2). Hemostasis could not be obtained with bipolar coagulation. Therefore, an additional assistant port was placed to enable gauze compression by an assistant surgeon, which achieved hemostasis after several minutes. We then decided to complete total mesorectal excision while compression hemostasis was performed. After mobilizing into the intersphincteric plane, the rectum was transected using the robotic stapler. Specimens were extracted via a mini-laparotomy at the umbilicus. Once uncontrollable bleeding thought to arise from the sacral vein occurred, we decided to convert to open surgery. Despite attempting several methods of hemostasis, we were not successful; total blood loss volume was 5.8 L and massive blood transfusion was required. Major bleeding was then controlled via gauze packing of the pelvic cavity. The abdomen was temporarily closed and the patient managed in the intensive care unit under complete sedation. The next day, a second-look operation was performed. After removing the gauze layers in order, all bleeding had completely subsided. To reinforce the hemostasis, a sheet of polygluconic acid was affixed to the sacrum and fibrin glue was sprayed (Fig. 3). An intestinal anastomosis was not performed in case of postoperative bleeding. The patient was extubated 2 days later and his postoperative course was generally good; however, a pelvic abscess developed and required percutaneous drainage. He was discharged on postoperative day 35. One year after the operation, he was free from recurrence. A colorectal anastomosis is planned.

**Fig. 4:**
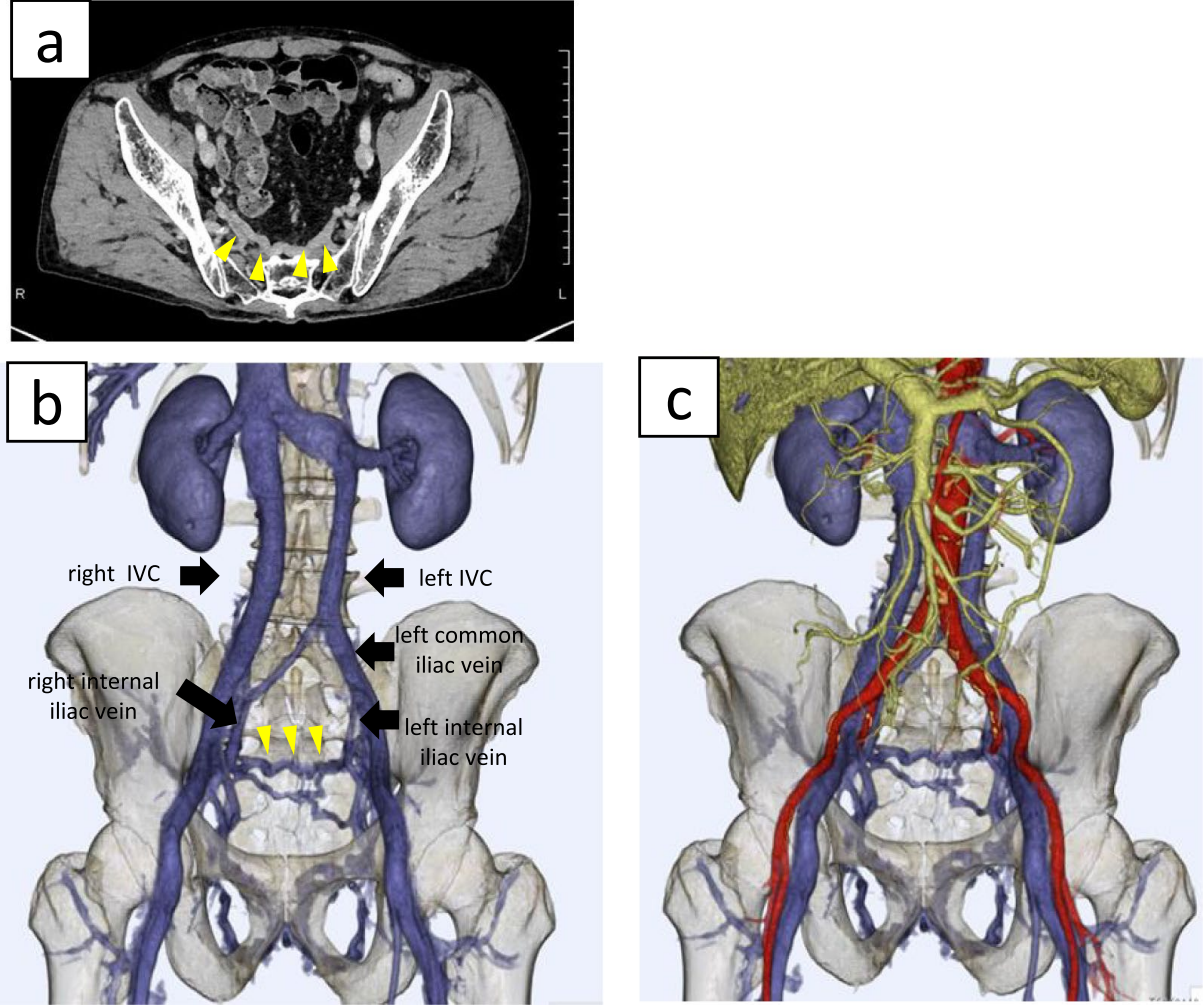
Pelvic computed tomography and three-dimensional computed tomography angiography. **a**, **b** A vein connecting the left and right internal iliac veins coursed anterior to the sacrum, forming an abnormal venous network (arrowheads). **c** 3D CT angiography of abdominal major vessels including portal vein, vena cava and abdominal aorta

In retrospect, the patient had a connecting vein between the left and right internal iliac veins which coursed anterior to the sacrum and formed a distended venous network. Also, venous anomaly was also seen in this case as follows; left IVC originated from the confluence of the left common iliac vein and a branch of the right internal iliac vein. Right IVC was mainly connected to the right external iliac vein, partially jointed to right internal vein (Fig. 4). This was presumably damaged during the operation, which caused the massive bleeding we encountered.

### Case 2

**Fig. 5:**
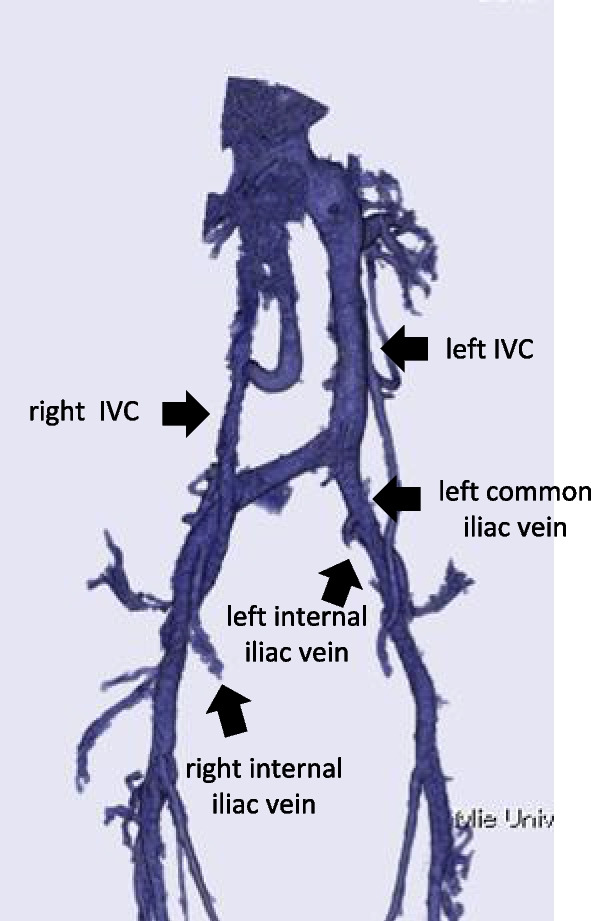
Three-dimensional computed tomography angiography of the inferior vena cava and pelvic veins. No abnormal veins are visualized anterior to the sacrum

A 64-year-old woman with lower rectal cancer and idiopathic thrombocytopenic purpura was referred to our department. Her platelet count was routinely below 5 × 10^4^/µL. On colonoscopic examination, her rectal cancer was located mainly in the lower rectum proximal to the dentate line. CT and MRI showed a DIVC and no enlarged lymph nodes or distant metastasis. Positron emission tomography/CT demonstrated no significant fluorodeoxyglucose accumulation outside of the primary tumor. Stage II lower rectal cancer (cT3N0M0) was diagnosed. Because of the DIVC, 3D CT angiography was performed for more detailed assessment (Fig. 5). Left IVC originated from the confluence of the left common iliac vein and the right common iliac vein. Right IVC was narrow and mostly connected to the right internal iliac vein, partially jointed to right external vein. Although such anomaly in vena cava to iliac vessels was identified, no noteworthy venous flow or anatomy was identified. Neoadjuvant chemoradiotherapy was deferred owing to her pre-existing thrombocytopenia. Therefore, short course radiotherapy (25 Gy delivered in five fractions) was administered followed by laparoscopic abdominoperineal resection without lateral pelvic lymph node dissection. After neoadjuvant treatment, the tumor size decreased from 30 to 20 mm.

After placing five ports, the medial approach was initiated, sparing the left ureter and gonadal vessels and both hypogastric nerves. The inferior mesenteric artery was resected at the root and D3 lymph node dissection was performed. During the medial to lateral approach, the left colic artery and inferior mesenteric vein were resected without visualizing the left IVC because of the retroperitoneal adipose tissue. In the pelvic phase, no aberrant arterial or venous anatomy was observed (as expected); therefore, the rectum was mobilized from the sacrum, pelvic splanchnic nerve, uterus, and vagina. After completing total mesorectal excision, the colon was transected intracorporeally using a laparoscopic stapler and the rectum was resected from the perineal side. The surgery was completed without complications. Duration of surgery was 270 min. Blood loss was minimal. The patient was discharged uneventfully.

## Discussion

DIVC is one of the most well-described IVC abnormalities. Most cases are detected incidentally because it rarely causes symptoms [1, 7]. DIVC forms in early embryonic development as a result of complex anastomoses and degeneration of the posterior cardinal, subcardinal, and supracardinal veins [8, 9]. Abnormalities in the evolution and involution of these veins can cause congenital anomalies. More specifically, persistence of both the left and the right supracardinal veins results in a DIVC.

Several studies have classified the complex anomalies that may occur during IVC development [1, 8, 10, 11]. Morita et al. classified pelvic venous variations into eight types using CT images of 36 patients with congenital IVC anomalies. Twenty-eight of 34 patients displayed double IVC. Of 28 DIVCs, 11 (39.3%) displayed no interiliac communication (type 2a), five (17.9%) displayed interiliac communication from the left common iliac vein (type 2b), one (3.6%) had communication from the right common iliac vein (type 2c), six (21.4%) had communication from the left internal iliac vein (IIV) (type 2d), and five (17.9%) had communication from the right IIV (type 2e) [11]. According to Morita's criteria, our two cases classified into type 2b, however, it might be exceptional that abnormal connection between bilateral internal iliac vein seen in case one. DIVC is accompanied by several abnormalities of pelvic veins [7, 11, 12]. Shin et al. [12] reviewed multi-detector computed tomography images of 2488 patients and reported that there were 40 cases (1.61%) of IVC abnormality and among them 23 patients (0.9%) had DIVC. They also showed that the prevalence of variations in the iliac veins is higher in patients with IVC abnormalities. Although few reports have examined abnormalities of the presacral venous plexus, Ito et al. reported abnormal connections between the right and left internal iliac veins, as seen in case 1 [7].

DIVC can pose a clinical problem in retroperitoneal and pelvic surgeries. Mao et al. reported a case of iatrogenic DIVC injury during radical nephroureterectomy and cystectomy in a patient in whom it was misdiagnosed [13]. When IVC anomalies are unrecognized, the incidence of fatal and uncontrollable bleeding approaches 10% [14].

Anterior resection of upper rectal cancer in patients with a DIVC has been previously reported [15, 16]; however, to the best of our knowledge, resection of advanced lower rectal cancer has not. In planning advanced lower rectal cancer resection, radiation therapy or lateral lymph node dissection should be considered to reduce local recurrence. When lateral lymph node dissection is performed, detailed knowledge of the iliac vein anatomy and possible variants is essential because it frequently requires manipulation of the internal and external iliac veins. Even if neoadjuvant radiotherapy is selected, radiation-induced fibrosis and exudate could increase the difficulty of surgery. In addition, when considering minimally invasive laparoscopic or robot-assisted surgery, in which the surgical field of view is limited, more detailed surgical planning is required.

In case 1, although DIVC was identified before surgery, we were not aware of the various risks that this anomaly poses. Difficulty in recognizing and mobilizing tissue layers owing to preoperative CRT might also have served as a risk factor. However, in case 2, detailed preoperative imaging was performed (3D CT angiography, MRI, and positron emission tomography/CT). As a result, we avoided serious complications related to the DIVC and pelvic venous abnormalities. To avoid these serious complications, DIVC and any associated anatomical abnormalities must be recognized before surgery to enable careful surgical planning.

## Conclusions

Patients with DIVC frequently also have other venous malformations that may become a problem when mobilizing the mesorectum from the retroperitoneum. In rectal cancer patients with a DIVC, preoperative assessment of pelvic vessel anatomy using 3D CT angiography is essential. Tumor stage guides the selection of appropriate neoadjuvant therapy and surgical procedure. Meticulous technique and surgical manipulation will contribute to safe performance of the operation, regardless of the surgical approach.

## Data Availability

Not applicable.

## Abbreviations

3D: Three-dimensional
CRT: Chemoradiotherapy
CT: Computed tomography
DIVC: Double inferior vena cava
IVC: Inferior vena cava
MRI: Magnetic resonance imaging
